# Replication Origins and Timing of Temporal Replication in Budding Yeast: How to Solve the Conundrum?

**DOI:** 10.2174/138920210791110942

**Published:** 2010-05

**Authors:** Matteo Barberis, Thomas W. Spiesser, Edda Klipp

**Affiliations:** 1 Institute for Biology, Theoretical Biophysics, Humboldt University Berlin, Invalidenstraβe 42, 10115 Berlin, Germany; 2 Max Planck Institute for Molecular Genetics, Ihnestraβe 73, 14195 Berlin, Germany

**Keywords:** Budding yeast, DNA replication, origins of replication, temporal program, stochastic firing, genomic instability, Clb5, Sic1.

## Abstract

Similarly to metazoans, the budding yeast *Saccharomyces cereviasiae* replicates its genome with a defined timing. In this organism, well-defined, site-specific origins, are efficient and fire in almost every round of DNA replication. However, this strategy is neither conserved in the fission yeast *Saccharomyces pombe*, nor in *Xenopus* or *Drosophila* embryos, nor in higher eukaryotes, in which DNA replication initiates asynchronously throughout S phase at random sites. Temporal and spatial controls can contribute to the timing of replication such as Cdk activity, origin localization, epigenetic status or gene expression. However, a debate is going on to answer the question how individual origins are selected to fire in budding yeast. Two opposing theories were proposed: the “replicon paradigm” or “temporal program” *vs.* the “stochastic firing”. Recent data support the temporal regulation of origin activation, clustering origins into temporal blocks of early and late replication. Contrarily, strong evidences suggest that stochastic processes acting on origins can generate the observed kinetics of replication without requiring a temporal order. In mammalian cells, a spatiotemporal model that accounts for a partially deterministic and partially stochastic order of DNA replication has been proposed. Is this strategy the solution to reconcile the conundrum of having both organized replication timing and stochastic origin firing also for budding yeast? In this review we discuss this possibility in the light of our recent study on the origin activation, suggesting that there might be a stochastic component in the temporal activation of the replication origins, especially under perturbed conditions.

## INTRODUCTION

In eukaryotic cells, DNA replication is restricted to a specific time window, called S phase. Successful progression through S phase requires replication to be properly regulated to ensure that the entire genome is duplicated exactly once, without errors, in a timely fashion, as any errors might generate chromosomal defects which can lead to genomic instability and death [1, 2]. As a result, DNA replication has evolved into a tightly regulated process involving the coordinated action of numerous factors that function in all phases of the cell cycle.

Eukaryotic replication starts from multiple locations referred to as replication origins throughout each chromosome. These sequences direct the formation of a number of protein complexes leading to the assembly of two bidirectional DNA replication forks at each origin. Research in the past years has identified many of the protein components of these complexes and the time during the cell cycle they assemble at the origin [1]. The density of active replication origins determines S phase dynamics and chromosome stability during mitosis [3], and in the budding yeast *Saccharomyces cerevisiae* a direct correlation between the length of S phase and the number of the replication origins has been demonstrated [4]. Experimental and computational studies have identified and mapped over 700 potential origin function target sites on the budding yeast genome [5-10] which are highly redundant. Despite the contribution that multiple origins per chromosome may give to an efficient genome duplication, direct experimental evidence indicates that yeast chromosomes have many more replication origins than they need for their timely replication during the S phase. In fact, several origins on chromosome III can be deleted without substantially affecting the ability to inherit this chromosome faithfully during cell division [11, 12]. Therefore, for the purpose of genome duplication, it is a challenge to investigate the relation between the number of active origins and the replication time, in particular whether a minimal origin set could be sufficient to complete DNA replication in the correct timing of the S phase.

Based on our recent study [13], we discuss in this review how origin firing could be globally coordinated to ensure an even distribution of replication initiation sites across the genome, highlighting the fact that in budding yeast, as for all eukaryotes, activation of the temporal program of origin activation can be driven by stochastic events.

## SPATIAL AND TEMPORAL REGULATION OF ORIGIN FIRING

Eukaryotic cells initiate DNA synthesis from hundreds of replication origins distributed over the chromosomes that constitute the genome. The temporal program of origin activation is imposed by well-defined cis-acting sequence features. The chromosomal sequences required for an origin of replication vary significantly between different eukaryotic organisms. In the unicellular eukaryote *S. cerevisiae*, three to four DNA sequences of 10–15 base pairs (bp) spread over 100–150 bp are sufficient to act as an origin and include the highly conserved and essential **A**utonomously **R**eplicating **S**equence (ARS) [14].

Efficient replication is guaranteed by multiple replication origins which are activated, or fired, per chromosome. For example, yeast chromosome III uses 11 origins for its duplication while chromosome X uses about 20 [5, 6, 15]. The budding yeast has a genome of 13.5 million base pairs (Mbp) distributed over 16 chromosomes, and each single chromosome is considerably smaller than the *Escherichia coli* genome of 4.6 Mbp. Yet yeast replication origins occur on average every 20–40 kb, a hundred times more densely distributed than one would predict by comparison to the *E. coli* genome. The difference in fork migration rates may explain in part the need for multiple replication origins per eukaryotic chromosome. DNA replication forks migrate at rates about 30 times slower in yeast (about 3 kb/min) compared to *E. coli* (about 100 kb/min) [5, 16]. The use of multiple initiation events per chromosome probably compensates for slower fork migration rates in maintaining an efficient rate of genome duplication and S phase progression in eukaryotic cells. Based on this reasoning, budding yeast would need about 100 replication origins to duplicate its genome at a rate sufficient to accommodate its S phase, about four times less than the current estimation [5, 6].

In reality, budding yeast has many more replication origins than necessary to complete the replication program. In fact, only a small number of origins seem to represent classic replicators, while the majority corresponds to zones of inefficient, closely spaced, start sites none of which are indispensable for origin activity. The fact that potentially only a subset of them is required to correctly replicate DNA according to a precise timing characteristic of the S phase could be related to several independent mechanisms that may affect the efficiency in origin firing or the timing of origin activation, including DNA sequence features intrinsic to the specific origin. Moreover, for many origins it is clear that temporal and spatial controls can contribute to the timing of replication modulating origin activity. The time during S phase when a chromosomal region is replicated correlates strikingly with chromatin structure and gene expression in many organisms, and transcriptionally inactive genes and/or heterochromatin are often the last portions of the genome to be duplicated [17-19]. A wide number of studies have established a role for cis-factors including chromosome context and chromatin structure in controlling the efficiency and timing of origin activation during S phase in *S. cerevisiae*, describing specialized chromatin-binding proteins, chromatin-modifying enzymes and chromatin remodelling proteins in controlling origin activity [20, 21]. Recently, a role of transcriptional activity on the replication timing has been discussed [22-24]. Moreover, different studies highlight the link between origin activity and origin localization [25, 26] and epigenetic factors regulating the time of firing [27-29].

## ORIGIN ACTIVATION AND REPLICATION INITIATION

DNA replication is regulated through interaction between cis-acting sequences and trans-acting initiation factors. Specifically, replication origins are determined by the binding, in late mitosis and early G1 phase, of a large complex of initiator proteins which assembly into well-defined complexes called pre-replicative complexes (pre-RC). This process is referred to “licensing”, and the pre-RC recognizes the origins and assembles to prepare them for firing. Formation of the pre-RC requires minimally the six subunit origin recognition complex (ORC), Cdc6, Cdt1 and Mcm2-7 [1, 30]. Subsequently, in early S phase, the pre-RC is converted into an active pre-initiation complex (pre-IC), with the inclusion of Cdc45, GINS (complex containing the four subunits Sld5, Psf1, Psf2 and Psf3, essential for both initiation and progression of DNA replication) and DNA polymerases, that facilitates unwinding the origins and starting the replication process [1, 30]. The process is summarized in Fig. (**1**). The activation of the replication machinery has still to be highlighted in many of its regulatory events, but a critical step is the availability of specific kinase activities at the G1/S transition. CDK (Cdk1-Clb5,6) and DDK (Cdc7-Dbf4) complexes phosphorylate components of the pre-RC setting the correct timing of S phase progression [31-39]. As shown in Fig. (**1**), in particular Cdk1-Clb5,6 activity exploits an essential role in the process, due to the fact that, remaining high from S to G2/M phases, it phosphorylates and, thus, negatively regulates ORC [40], Cdc6 [41-45], Cdt1 and Mcm2-7 [46-49]. The effect of these phosphorylations is to prevent the assembly of new pre-RCs until the next G1 phase [50] and differs from protein to protein, as ORC is inactivated while Mcm2-7 and Cdt1 are exported from the nucleus, and Cdc6 is degraded. In addition, the physical interaction between Clb5 and ORC helps to inhibit re-initiation [51]. Cdk1-Clb phosphorylation of the initiation factors Sld2 and Sld3 is also a critical step for the initiation of DNA replication [34-37].

## ORIGIN ACTIVATION BY CLB-DEPENDENT CDK ACTIVITY

Several studies have suggested that yeast chromosomes contain early and late replicating domains and exhibit replication timing profiles that are consistent with a highly regulated chronological program [7, 9, 52], favoring the argument that, differently from other eukaryotes, in budding yeast the origins of replication fire according to a defined temporal program [53]. Each individual origin is characterized by both a specific reproducible time of activation and a firing efficiency during the replication process in the S phase of cell cycle [54]. Generally, early origins are positioned toward the central portion of chromosomes, while late origins are positioned near the telomeres such that the central portion of chromosomes replicate before the ends [55, 56]. Detailed data are available for yeast chromosome VI [57, 58]. However, there is not a consistent relationship between replication origin efficiency and the time at which an origin fires during S phase, being some late firing origins efficient and others not (data for chromosomes VI and XIV are reported [58, 59]). Some late origins are inefficient because they are located near an earlier firing origin, which replicates the later origin before it has a chance to fire [60, 61]. Thus, competition between two closely spaced origins can influence origin efficiency and, consequently, the replication profile of origin activation [62].

Regulation of the S phase program occurs at several stages, affecting origin firing, replication fork elongation, fork velocity and fork stability, all dependent on S phase-promoting kinase activities. Origin selection is in fact synchronised with cell cycle progression by coupling origins that are activated preferentially during early or late S phase to the expression of specific Cdk-cyclin complexes. In mammalian cells, initiation of early origin firing is regulated by the trans-acting factors Cdk2-cyclin A or Cdk2-cyclin E kinase activities. In addition, Cdc7 is also reported to be involved in the regulation of entry into S phase. These two kinases are involved in the loading on origins of Cdc45 by phosphorylating subunits of Mcm2-7 complex [63]. Transfactors that regulate late origin firing have been suggested as well, where Cdk1-cyclin A2 has been proposed to have this essential role [64]. Early or late firing of the origins during S phase accounts for much of the temporal programming of replication in budding yeast, and specific Cdk-cyclin complexes play a dominant role in defining the origins that are active during early and late S phase. Cdk1-Clb6 plays the major role in early origin activation, before the degradation of Clb6 during S phase [65]. Cdk1-Clb5 is known to be indispensable for activation of late replication origins, showing that *clb5Δ* cells failed to activate these origins and subsequently showed an extended S phase [52, 66]. Interestingly, this late origin firing defect is suppressed although entry into S phase and is significantly delayed in *clb5Δ* *clb6Δ* cells, suggesting that other B-type cyclins promote firing of both early and late replication origins.

There is a strong interest in understanding mechanisms of origin selection for the proper replication timing. This temporal program of replication refers to the propensity for different regions of the genome to be replicated at different times during S phase, resulting in a temporally regulated origin activation [67]. However, despite the fact that quite some details controlling the activation of the replication origins have been elucidated – especially concerning the role of kinase activities – and that a temporal program of the S phase progression must be a fundamental feature to ensure a precise DNA synthesis, the mechanisms underlying the observed pattern of replication remain unknown.

## TIMING IN ORIGIN ACTIVATION: TEMPORAL PROGRAM OR STOCHASTIC FIRING?

Since molecular mechanisms underlying the regulation of origin activation or origin firing are strongly conserved across evolution from budding yeast to human cells [1], unicellular model organisms have been chosen to investigate the mechanisms underlying the regulation of replication origins and the temporal program of replication. In particular, patterns of replication timing have been studied in *S. cerevisiae* [5, 52, 55, 68, 69], with the advantage that the location of origins is mapped through the whole genome and the average firing time of each origin has been calculated [5, 7]. These studies show that origins in budding yeast fire at characteristic times, with some origins firing on average earlier and others firing on average later. Budding yeast has well-defined, site-specific, origins many of which are efficient and fire in as many as 90 % of S phases [70, 71], and it is apparently more close to the mechanism of bacterial replication, in which origins are well-defined sites that fire once in each cell cycle, leading to uniform replication that is identical in every cell [72]. On the other side, in fission yeast and vertebrates, less than 50 % of potential origins fire in each S phase [8, 73-78]. This inefficient origin firing leads to an asynchronous initiation of DNA replication throughout S phase at indiscriminate sites and a different subset of origins firing in each cell, causing a random pattern of replication in each cell cycle. The excess origins may serve as backups to ensure efficient replication if origin firing is compromised or replication forks stall [79-82]. Interestingly, recent studies have revealed an intrinsic temporal disorder in the replication of yeast chromosome VI [83], suggesting that also stochastic events could influence the replication program, as it is the case for the fission yeast *Schizosaccharomyces pombe*, *Drosophila melanogaster* and *Xenopus laevis*.

Stochastic events related to the firing of specific origins can possibly determine a different pattern of origin activation. The earlier origin establishes a replication fork that replicates the later origin before it has a chance to fire [61], and direct experiments support a role for such an “origin interference” mechanism in origin efficiency [84-86]. Presumably, this inactivates the competent initiation complex assembled at the late origin [6], despite studies have shown that the assembly of this protein complex occurs – regardless of their initiation time – at all origins during mitosis [87, 88]. The reproducible replication times observed for origins in budding yeast were interpreted to demonstrate that origins fire at predetermined times in S phase [5, 7]. However, these analyses assay the average behavior of individual origins over a population of cells and it is possible that replication timing is heterogeneous on a single-cell level. Thus, a peak in a replication profile indicates a firing of an origin at a specific chromosomal location, but because the profile is generated from a population of cells, it is not possible to know whether a specific origin fires in every cell in the population [89, 90]. Czajkowsky and colleagues demonstrated heterogeneous patterns of origin firing suggesting that replication origins fire inefficiently and stochastically, instead of firing efficiently and at defined times during S phase. Moreover, replication origins show a wide efficiency, some firing in almost every cell cycle while others fire in less than 10 % of the cells [5, 58, 59]. Strikingly, the replication profiles observed by Czajkowsky and colleagues were similar to the previous ones obtained from microarrays experiments [5, 7, 91]. This comparison demonstrates that stochastic firing is compatible with defined replication timing [90].

Origin properties, deterministic (chromosomal position and firing time) and stochastic (firing efficiency), possibly influence DNA replication. In particular, the efficiency of an origin has a stochastic influence because it changes the shape of the replication profiles representing activation of the origins (the more efficient ones) in time. However, replication profiles discard much of the information from the experiments and cannot distinguish late, efficient origins from early, inefficient origins. Moreover, an observed firing time does not necessarily represent the time at which a replication origin fires in S phase, but reflects the fact that an origin that replicates late in S phase could do this either because it fires efficiently late in S phase or because it fires early but is inefficient and, thus, passively replicated later in S phase. As illustrated in Fig. (**2**), this results in different landscapes observed for an origin activation because an origin will not necessarily fire in every cell cycle, being characterized by a variable firing competence, i.e. the percentage of cells in which an origin is biochemically competent to fire [89, 90]. If it does, the origin is considered to be efficient, having a high competence to fire; if it does not fire regularly, then it is called inefficient, being characterized by a low firing competence. In the example reported, the arbitrary replication profile shows that origin number 2 has a high competence of firing whereas origin number 5 has a low competence.

The bulk of evidence presented suggests that possibly neither of the two mechanisms of replication, the temporal program of origin activation and the stochastic firing, reflects the reality of replication in budding yeast. Conversely, a combined effect of both could represent it [89, 90]. It has been proposed that an alterative model based on the stochastic firing of origins may also explain replication timing, assuming varying origin efficiency instead of a strict origin-timing program. So how can these apparently conflicting data be reconciled to explain the replication pattern reported experimentally [5-7]? As nicely discussed by Rhind in 2006, stochastic firing of origins leads to the so-called “random gap” problem: randomly distributed origin firing will occasionally lead to large gaps between replication bubbles that would take a long time to replicate [53]. To reconcile the random distribution of origin firing with efficient replication, it has been proposed that origins can fire stochastically – when they fire, the firing times are maintained – but the efficiency of origin firing increases as S phase progresses [53, 90]. Therefore, the longer a large random gap persists, the more likely origins within it are to fire. This has been firstly proposed for *Xenopus* embryos [79, 92]. This approach was already faced by Takahashi in 1987, where a model for the spatiotemporal organization of DNA replication in mammalian cells was generated [93]. It could account for a partially deterministic and partially stochastic order of DNA replication in chromosomes, giving the possible solution to reconcile the apparently different characteristics of having both organized replication timing and stochastic origin firing.

## DETERMINISTIC MODEL OF REPLICATION DYNAMICS IN BUDDING YEAST

The distinction between deterministic program of origin activation and stochastic firing is only an apparent conflict. Biology is inherently stochastic, and the question that has been pointed out is not whether origin firing is stochastic, but how important is the probabilistic nature of origin firing in the regulation of replication timing [90]. More importantly, how can stochastic origin firing be accommodated in realistic models that predict the patterns of replication timing observed *in vivo*? The proposed deterministic/stochastic approach to investigate the replication program considering the increasing efficiency of the replication origins during the DNA synthesis is, at the moment, not really applicable for budding yeast due to the availability of few data about individual origin efficiencies [58, 59]. In this case, a fully stochastic model could be developed to explore the temporal program of origin activation, as it has been proposed both for fission yeast [94] and *Xenopus* embryos [95]. Modeling approaches are, at least initially, most fruitful in simple systems where more of the parameters are known, and *S. cerevisiae* represent a good example of study because quite an amount of data are available about the replication origins. For this unicellular organism, origin location of replication origins on the chromosomes [14] and initiation time of origin firing for a subset of replication origins [5, 7] are reported in the *S. cerevisiae* OriDB database [54], and replication fork rate values are available [5, 7, 16].

To date, no mathematical models investigating the temporal activation of the origins in budding yeast are available, thus we used the accessible information to generate the first, fully deterministic, mathematical model of DNA replication in *S. cerevisiae* in order to investigate the temporal sequence of origin activation [13]. We used the length of the chromosomes, the position of selected replication origins, their initiation time and a fork migration rate assumed to be constant to recalculate the replication profiles of all 16 chromosomes, assuming that the efficiency of the selected origins was 100 %. Specifically, two studies report the timing for different replication origins, but they are not consistent if compared to each other. In the first study, Raghuraman and colleagues report the timing for 454 origins [5] whereas, in the second one, Yabuki and colleagues provide such information for 260 origins [7]. Considering that the first study provides information for a bigger subset of replication origins, we included these data in our study. We wanted to test whether the assumptions considered to build the deterministic model were acceptable. Essentially, we: (i) considered both chromosomal location and firing time derived from the microarray-based heavy::light (HL) analysis for 454 replication origins (~ 60 % of the total origins – 732 – included in the *S. cerevisiae* OriDB database), (ii) assumed that replication forks migrate constantly throughout S phase at an approximate rate of 3 kb/min, averaging the experimental data available (mean of 2.9 kb/min and a median of 2.3 kb/min) and (iii) assumed an efficiency equal to 100 % for the 454 origins (this corresponds to a single replication event in a cell with 732 origins that fire with an at efficiency of about 60 %).

As shown in Fig. (**3**), in the deterministic model the DNA is divided into units (*u*) of equal length (500 bp). Hence, in the simulation each chromosome is composed of a series of DNA units, corresponding to its original size (*L_org_*) divided by 500 to yield the internal resolution size. A two-dimensional array element (*A*) of size *L_res_* is assigned to every chromosome, and two DNA units are added to account for the left and right end of the chromosomes. The array element *A* contains all discrete DNA unit positions and the status of the replication for a specific position. This is represented by a Boolean variable, which is set equal to 0 when the DNA unit has not been replicated at this position yet, and set to 1 when the DNA unit has been replicated. Another two-dimensional array element stores origin information: origin name, origin position on the virtual chromosome *A*, origin activation time in seconds and the origin activation status, a Boolean variable set to 0 by default, indicating that the origin has not been activated yet. A variable *T* represents the replication time. Each of the discrete time steps needed to complete DNA replication is equal to the time (Δ*t*) that the replication fork needs to process one DNA unit (Δ*u*): Δ*t* = Δ*u* / Δ*v*, where Δ*u* = 500 bp and Δ*v* = 3 kb/min and, therefore: Δ*t* = 10 sec. The simulation proceeds in the way that at each time point *T_j_* the algorithm reviews the array to find the origins that fire at that time. If so, the Boolean variables in the array *A* for these origins are set to 1, indicating that they have been activated and cannot fire anymore. Every origin issues two replication forks upon activation, each travelling in opposite directions on the DNA and therefore, at each time point *T_j_*, the algorithm checks whether the positions left and right of a replicated region have been replicated or not. In this way, replication forks migrate in both directions until they encounter either a region that has been already replicated or the end of the chromosome. During the simulation every replication fork through the genome can be retraced and their final positions and times can be mapped.

In spite of the approximations on which we generated the model, the recalculated replication profiles of origin activation matched the experimental profiles surprisingly well for 10 out of 16 chromosomes (see [13] for detailed comparison between computed and experimental replication profiles obtained from Raghuraman and colleagues). However, significant differences were observed for the other 6 chromosomes, with many sequences replicating later than predicted. One possible explanation is that whereas the slope of the recalculated profiles is constant due to the constant fork rate implemented, the experimental curve is smooth with a varying slope, reflecting a changing of the fork progression rate – due to the folded structures of the DNA or binding of specific protein factors to the origins which drive accessibility and motion of the polymerase – or the activation of inefficient origins that were not included, as also highlighted out from Hyrien and Goldar [96].

## FROM A DETERMINISTIC MODEL TO A STOCHASTIC OUTPUT

The identified and mapped potential origins of replication on the genome of budding yeast [5-10] are redundant. Due to the intriguing fact that excess of origins may ensure efficient replication if origin firing is compromised [79-82], we investigated the relation between the number of active origins and the replication time.

By using our deterministic model, we assessed the impact of particular sets of origins on the replication time computing the replication kinetics in which the DNA is duplicated in time for all 16 yeast chromosomes. We obtained the classical “switch” for the origin activation, characteristic of the prompt availability of the Cdk1-Clb5,6 activity at the entry into S phase of the cell cycle [97]. Both Clb5 and Clb6 are essential in this process, and activate different subsets of replication origins to complete the replication program [52, 66, 98]. Then, we computed replication kinetics in perturbed conditions, i.e. considering different subsets of origins randomly deleted for 50 % of the total number used in the study (454), to simulate the wild type, mimicking in this way stress conditions, inefficient firing or checkpoint activation due to possible damage to the DNA and stalling of the replication fork [99]. Intriguingly, the perturbation showed only a small influence on the replication kinetics (delay in the termination of the replication), indicating that the precise timing of DNA replication was apparently not depending on specific origins potentially lost in the random deletion [13]. This means that DNA replication is robust against failure of origin firing or changes in origin efficiency, suggesting that the DNA could be duplicated through S phase independently from the temporal program of origin activation.

So, why having a temporal program if it is apparently not needed? Is this the pattern of evolution from a fix, deterministic program to a redundant, stochastic process? The result previously obtained stimulated us to further test the spatiotemporal model for understanding the relation between origin activation and replication time. We computed the time of the replication kinetics for each chromosome decreasing randomly the number of active replication origins, mimicking how the replication time could change in the case that a certain percentage of the origins would be deleted, defective or inefficient. As logically expected, systematically deactivating an increasing number of origins (from 10 % to 90 %) yielded an increasing time needed to complete DNA replication. However, strikingly, this analysis showed that for many chromosomes the replication times measured experimentally can be obtained using only subsets of activated origins [13], which are different for every chromosome and composed randomly.

The analyses performed suggest that in budding yeast a temporal program of origin activation might be not enough to describe all events occurring during replication, revealing how stochasticity in origin usage confers robustness and reliability to the DNA replication process. If the temporal activation of the origins is influenced by stochastic events that select randomly the origins, the process of DNA replication could be more efficient when perturbations affect the normal state of the cells. Considering that the density of active replication origins determines S phase dynamics [3], wheather the observed robustness of the replication is maintained also *in vivo* is an essential task to be proven. Experimentally, this feature might be tested by progressively deleting the known replication origins and evaluating the consequent replication time for each chromosome. Moreover, in the light of this evidence, the deterministic model could be now suitable to include values for origin efficiencies to test whether an increasing efficiency of firing through S phase could increase the reproducibility of the replication profile experimentally observed [5]. Since few data are available about origin firing efficiency [58, 59], and more have still to be produced, a model which considers an *in silico* progressive increase of the efficiencies could be developed, to generate a partially deterministic and partially stochastic system in order to investigate whether this is the proper strategy to solve the “random gap” problem [53].

## ROLE OF CDK1-CLB ACTIVITY IN MATHEMATICAL MODELS OF DNA REPLICATION: TIMING OF ORIGIN ACTIVATION

Entry into S phase of the cell cycle is driven by the B-type cyclin (Clb)/cyclin-dependent kinase (CDK) complexes Cdk1-Clb5 and Cdk1-Clb6 [97], the activity of which plays an important role in the control of replication timing, being a direct activator of origin firing. As previously described, Cdk1-Clb5 can activate both early and late origins, whereas Cdk1-Clb6 is only capable of activating early origins of replication [52, 66, 98].

We have recently investigated computationally the role of the Cdk1-Clb activity in the activation of the replication origins, compiling a huge amount of experimental data available in the literature and modelled the G1/S transition in *S. cerevisiae* [100]. The mathematical model was implemented by ordinary differential equations (ODEs) taking into account both nuclear and cytoplasmic compartments. Model tests revealed the main known regulatory events that impinge the functionality of this window of the cell cycle. In this study, we correlated the main output of the model, the nuclear concentration of Cdk1-Clb5,6, with the temporal activation of the replication origins and, thus, the onset of DNA replication. Considering the large number of replication origins present in a yeast nucleus [5-7] and the reported role of Cdk1-Clb5,6 in inducing firing, we associated the probability of firing for each replication origin to the nuclear concentration of Cdk1-Clb5,6. The influence of Cdk1-Clb5,6 on the licensing of replication origins has been described by a probabilistic three-step model as represented in Fig. (**1**), which does not consider molecular details of this highly regulated process [100, 101], and the distance between the considered average of replication origins (440) has been fixed. Step 1 lumps all events from free origin on the DNA to the pre-RC formation. The transition time for each of the replication origins considered was taken from a normal distribution with mean of 15 min and standard deviation of 2 min. The probability for performing Step 2 at a certain time is determined by the concentration of nuclear Cdk1-Clb5,6 at that time. The duration of this step is necessary for Cdk1-Clb5,6 to exceed a value taken from a normal distribution with a mean of 0.03 μM and standard deviation of 0.01 μM. The transition time for Step 3 to reach the “fired” state of a replication origin was again taken from a normal distribution with a mean of 1 min and standard deviation of 0.01 min. When an origin has fired, then DNA replication proceeds bidirectionally from multiple replication origins, as experimentally reported [102, 103]. If the replication reaches the neighboring origin before it fires on its own, that origin is set to the state “fired”. The probabilistic model of the origin activation successfully explains the activation of DNA replication (i) in cells grown in different nutritional conditions (glucose and ethanol media) and (ii) of different yeast background, i.e. single deletion or overexpression mutants of central players at the G1/S network [101]. This supports the fact that the Cdk1-Clb5,6 activity sets the basis for the prompt starting of the DNA replication machinery.

In addition, we were interested to study in more detail the role of Clb5 in activating the origins by our spatiotemporal model of DNA replication [13], in order to test whether our computational tool could be useful to reproduce the chronological program of origin activation, which has been shown to be reproducible even under altered conditions [91]. In fact, it has been demonstrated that *clb5Δ* mutant suffers a significant decrease in firing efficiency of the late origins in S phase [66]. Investigating the activation of replication origins in the *clb5Δ* mutant, McCune and colleagues pointed out that DNA replication in budding yeast follows a regular temporal program of origin activation rather than a disordered firing [52] and this evidence suggested us to look into the deterministic model of origin activation to test the reproducibility of the experiment. To this purpose, we divided the replication origins in an early (Clb5-unaffected) and in a late half (Clb5-affected) by stopping origin firing at the time corresponding to the mean value of the distribution of the experimentally determined origin firing times (27 min, about the midpoint of a normal S phase) [5, 13]. The replication profiles computed for the 16 yeast chromosomes in the *clb5Δ* mutant show the general agreement of the replication kinetics between the computed profiles and the experimental ones [13, 52]. The chromosomal portions suffering significant delays in replication correspond to the CLB5-dependent regions (CDRs) reported by McCune and colleagues, as illustrated in Fig. (**4**). In detail, we found a perfect match of the *in vivo* data for chromosomes I to VIII and XI, a good fit in the majority of the sequence length for chromosomes IX, X and XIV, and a very poor match for chromosomes XII, XIII, XV and XVI. Intrinsic noise might affect the firing time of the Clb5-dependent origins, therefore the considered value of about 27 min is an approximation, which for some chromosomes could be quite accurate, but for others it could be not the case. This might result in the fact that the chromosomes containing more early origins would be less sensitive to *CLB5* deletion, whereas the chromosomes with more late origins would be more sensitive. However, this analysis is in agreement with the fact that the *clb5Δ* mutant only affects late origins, whereas the early origins fire normally, and indicates that our deterministic model is indeed able to reproduce the temporal program of origin activation [13, 52].

Altogether, the results presented here suggest that our deterministic spatiotemporal model for DNA replication in budding yeast is able both to reproduce the chronological program of activation of the replication origins and to capture possible stochastic events in their temporal firing. This feature has been never considered before and, for the first time to date, we propose that also for budding yeast the probabilistic nature of origin firing might affect the regulation of replication timing. Due to the high degree of conservation of the DNA replication process from yeast to higher eukaryotes, this study opens the possibility to study the regulation of the origin activation introducing specific components whose deregulation is often fatal and can lead to severe genetic disease and cancer in humans.

## REGULATING CDK1-CLB ACTIVITY PREVENTING GENOME INSTABILITY

The evolution of multiple mechanisms to prevent DNA re-replication by the Cdk1-Clb5 activity clearly indicates the importance of avoiding this type of aberrant condition, which in humans has been associated with genomic instability and cancer [104]. The concept of genomic (or genetic) instability refers to a series of chromosomal changes occurring at an accelerated rate in cell populations [105] and indicates the increased tendency of tumor cells to acquire new mutations with each cell division. The importance of maintaining normal levels of origin efficiency during DNA replication is suggested by the fact that both increasing and decreasing origin efficiency correlates with decreased genomic stability, as it has been demonstrated both in mammalian [106] and yeast [107] cells. Increasing of origin efficiency could be caused by a diffusible, rate-limiting activator, such as CDK or DDK kinases required throughout S phase for origin firing [1, 30]. If one of them is rate limiting, this would limit the efficiency of origins during S phase. At first, the number of firing origins would be a small faction of potential origins, but as S phase progresses and the number of potential origins declines, that number would become an ever larger fraction of potential origins and the efficiency of the remaining origins would increase [90]. Other regulators of origin firing, such as the stable fork component Cdc45 or the mini-chromosome maintenance complex Mcm2-7, could also function as rate-limiting activators. For example, in mammalian cells, Cdc45 can recruit Cdk2 at the replication foci [108] and such interaction may explain the correlation between fork density and origin firing [95], although it is unknown whether it is conserved in yeast. Moreover, both in budding yeast and higher eukaryotes, the putative helicase Mcm is present in a large excess compared to the amount needed to replicate the genome [79, 109, 110]. Some of that excess Mcm is loaded at origins that will not fire, but some of the excess is loaded as multiple Mcm complexes at individual origins [111, 112]. Thus, the efficiency with which ORC loads Mcm at a given origin, or the amount of time ORC is bound to an origin and able to load Mcm, could determine an origin’s firing probability [90].

Both in yeast and in higher eukaryotes, genomic instability often ensues when the G1/S transition of the cell cycle is deregulated and cells are forced to enter S phase prematurely. This acquired mutability is important since a majority of genes mutated in human cancers influence the G1/S transition [113]. In *S. cerevisiae*, a central regulatory component that controls Cdk1-Clb5,6 activation at the G1/S transition is the stoichiometric Cdk-Clb inhibitor Sic1 [114-117]. The initiation of DNA replication is regulated by an irreversible switch in which Sic1, the functional and structural homologous of the CDK inhibitor p27^Kip1^ in mammalian cells [118], is multi-phosphorylated during late G1 phase by the G1 cyclin (Cln)/cyclin-dependent kinase (CDK) complexes Cdk1-Cln1,2 [119, 120] and, then, degraded at the G1/S transition by the ubiquitin/proteasome pathway before initiation of DNA replication [121-123]. Accumulation of active Cdk1-Clb5,6, as well as of the other Cdk1-Clb complexes active from S to M phases, enhances Sic1 destruction through the same mechanism and advances cells into S phase [124]. Sic1 is important for maintenance of a Cdk1-Clb-free windows of time which is critical for origin licensing. *sic1Δ* mutant initiate DNA replication from fewer origins, S phase initiates early and is extended compared to wild type, but mitosis is not delayed [107]. Moreover, chromosome combing experiment showed that on average the distance between replicons is 1.5 times longer in *sic1Δ *cells compared to wild type [125]. As a consequence, chromosomes break and rearrange at a very high frequency, and cells exhibit about a 100-fold increase in minichromosome loss and gross chromosomal rearrangements (GCRs) compared to wild type [107, 126]. This clearly shows that origin density is crucial for genome integrity. The precocious Cdk1-Clb5,6 activation causes chromosome rearrangements and severe genome instability through its inhibitory effect on pre-RC formation in late G1 phase. Thus, by inhibiting any residual Cdk1-Clb activity in G1 phase, Sic1 promotes efficient pre-RC formation, probably also on sites that serve as dormant origins [127].

With the purpose to investigate genomic instability in budding yeast, we recently modelled the network of the G1/S transition analyzing the implications of the balance between Cdk1-Clb5,6 and Sic1 for cell cycle progression [100] and studied the efficiency of this control in activating the replication origins at the entry into S phase [101]. According to the current scenario reported in literature, Sic1 is involved in the control of DNA replication as a negative regulator of the Cdk activity, and the mathematical models of the cell cycle of course are taking into account only this function [128]. This leaves unanswered a major phenotype of the *sic1Δ *mutant, namely sparse origin firing [107]. Thus, considering that Sic1 acts not only as a stoichiometric inhibitor of the Cdk1-Clb complexes [115, 118] but also as a promoter of Cdk1-Clb5,6 entry into the nucleus [100], as shown experimentally [129], we gave a rationale explanation for the sparse origin firing phenotype observed in the *sic1Δ *mutant [107]. As shown in Fig. (**5**), we observed an early firing of the replication origins in the mutant compared to the wild type, since no Sic1 degradation is required and Cdk1-Clb5 activity can fire the origins as soon as it becomes available, and proceeds slowly, as experimentally observed [107]. These results strongly suggest that the fine tuning of the Cdk1-Clb5,6 activity by Sic1 controls the precise temporal activation of the replication origins for the correct completion of S phase events. Whether the role of Sic1 in regulating origin firing is exploited only through inhibition of the Cdk1-Clb activity or in addition *via *direct binding to- or regulation of- the components of the DNA replication machinery has still to be investigated.

The fact that cells replicating their chromosomes from a sub-optimal number of origins are karyotypically unstable is highly important for the understanding of tumorigenesis, consistent with the prevalence of G1/S regulator mutations in cancer. The mathematical approaches here presented provide useful tools to implement components which deregulation drives an abnormal replication dynamics and to predict further scenarios in the temporal activation of the replication origins which in turn can be tested experimentally.

## CONCLUSIONS

Plasticity is an inherent feature of chromosomal DNA replication in eukaryotes. In budding yeast, origins of replication are made in excess and are potentially activated in a partly chronological programmed order and partly stochastically throughout the S phase of the cell cycle. In addition, origin activity is influenced by accessibility of replication origins, availability of regulatory proteins, chromatin structure and epigenetic factors, which contribute to the observed timing of replication. Since mathematical analysis explaining the temporal activation of the replication origins in budding yeast is still not available, our probabilistic/deterministic models of DNA replication dynamics are the first tool enabling to address this question and to suggest that stochastic events can influence the profile of the replication program.

## Figures and Tables

**Fig. (1):**
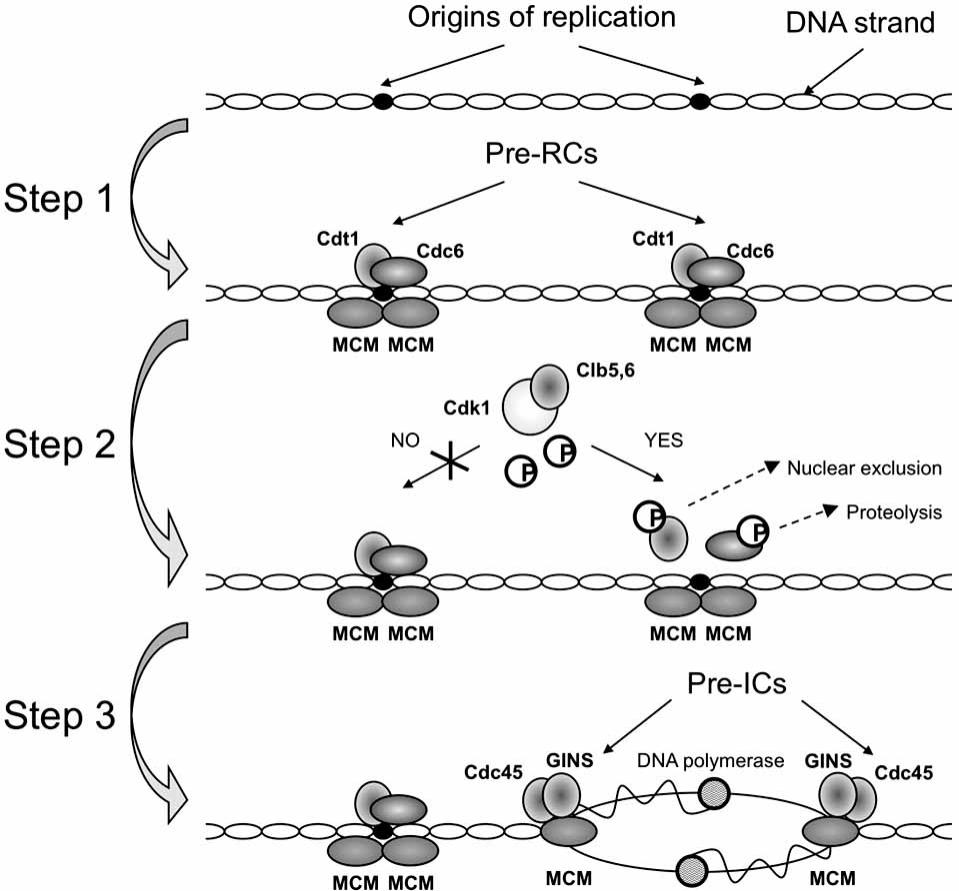
**Schematic representation of the DNA replication process.** Pre-RC components are recruited to the replication origins in a stepwise manner during late M and early G1 phases of the cell cycle. At the origins, ORC recruits Cdc6 and Cdt1, which in turn are required for the subsequent loading of the Mcm2–7 complex. After pre-RC has assembled, its activation during S phase involves the assembly of a second set of factors like Cdc45 and GINS as well as the activity of Cdk1-Clb5,6, which phosphorylates and determines the fate of specific components of the replication machinery. Origin firing and DNA replication then start bidirectionally (modified from Barberis and Klipp, *Genome Inform*. 2007).

**Fig. (2):**
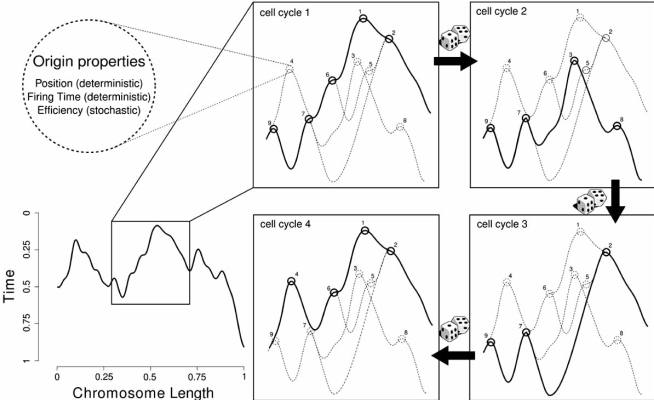
**Stochastic events influence origin activation and replication profiles.** An exemplified replication profile, i.e. the graph of when during S phase each locus in the genome is, on average, half replicated, is presented. In the replication profile, origins are peaks and peak height correlates with origin timing and efficiency. Focusing on the stochastic processes that can impinge the landscape of origin activation in four consecutive rounds of DNA replication, each panel represents a specific pattern of origins firing (solid lines) that can be observed out of the all possible firing landscapes (dotted lines). Replication origins are numbered from 1 to 9 according to the increasing firing times. Considering a competence of firing of an origin equal to 100 % if it is activated over four rounds of replication, the competence will be reduced in the case that the origin fires in some but not in every cell cycle: 75 % (origins 2, 7 and 9), 50 % (origins 1 and 6), 25 % (origins 3, 4 and 8) and 0 % (origin 5).

**Fig. (3):**
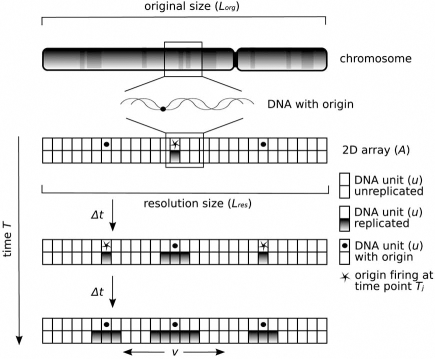
**Scheme of the deterministic model of DNA replication and its parametrization.** The features and the algorithm are explained in the text (reproduced with kind permission from Springer Science+Business Media: *Mol. Genet. Genomics*, A model for the spatiotemporal organization of DNA replication in Saccharomyces cerevisiae, 2009, *282*, 25-35, Spiesser, T.W., Klipp, E., Barberis, M., Fig. (**1**), and any original (first) copyright notice displayed with material).

**Fig. (4):**
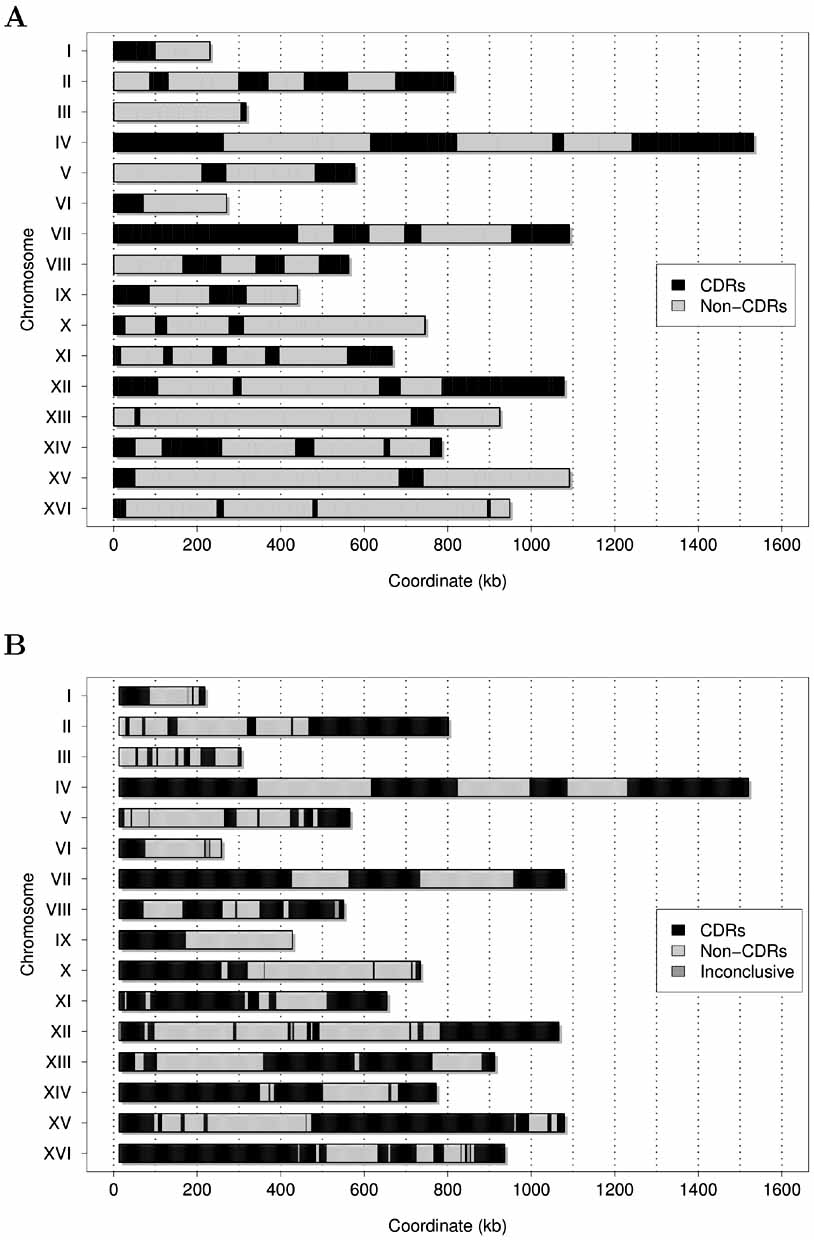
**Computed Clb5-dependent regions in the budding yeast genome.** The computed profile for the *clb5Δ* mutant is reported, showing the organization of the genome in Clb5-dependent and -independent regions (CDRs and Non-CDRs, respectively). CDRs are visualized in black and Non-CDRs in gray. The genomic regions of the CDRs for the 16 yeast chromosomes generally correspond to the ones identified by McCune and colleagues [37]. In detail, a perfect match is found for nine chromosomes (from I to VIII, and XI), a good fit for chromosomes IX, X and XIV, and a small or no match for chromosomes XII, XIII, XV and XVI [13] (compare Fig. (**2**) and Fig. (**4**) in [37]).

**Fig. (5):**
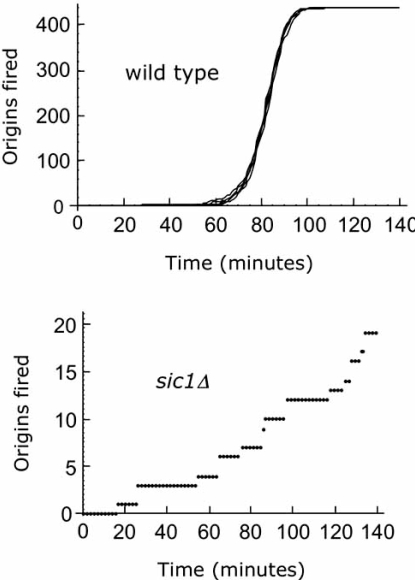
**Simulation of origin firing for wild type and *sic1Δ* cells**. The cumulative number of fired origins was calculated basing on the probabilistic model for firing of origins in wild type cells (upper panel) and the *sic1Δ* mutant. To be noted the different scales on the y-axis.
